# Factors Associated with Surgical Outcomes after Bilateral Lateral Rectus Recession in Children with Intermittent Exotropia

**DOI:** 10.3390/jcm13030731

**Published:** 2024-01-26

**Authors:** Seung-Ahn Yang, Hee-Young Choi, Su-Jin Kim, Kwang-Eon Han, Ji-Eun Lee

**Affiliations:** 1 Department of Ophthalmology, Pusan National University Yangsan Hospital, Pusan National University School of Medicine, Yangsan 50612, Republic of Korea; 2 Research Institute for Convergence of Biomedical Science and Technology, Pusan National University Yangsan Hospital, Yangsan 50612, Republic of Korea; 3 Department of Ophthalmology, School of Medicine, Pusan National University, Busan 46241, Republic of Korea; 4 Biomedical Research Institute, Pusan National University Hospital, Busan 49241, Republic of Korea

**Keywords:** intermittent exotropia, part-time patching, surgical outcomes

## Abstract

**Backgroud**: To analyze the factors associated with surgical outcomes after bilateral rectus recession (BLR) in children with intermittent exotropia (IXT). **Methods:** A retrospective study was performed on 125 patients who had all received preoperative patch treatment with a ≥1 year follow-up. The surgical outcomes were grouped as success (esodeviation ≤5 PD to exodeviation ≤10 PD) or failure (esodeviation >5 PD or exodeviation >10 PD) according to the angle of deviation at 1 year postoperatively. The patients’ magnitude of exodeviation, near and distant stereoacuity, and 3-mo patch responses were assessed. The factors associated with the surgical outcomes were determined using univariate and multivariate analyses. **Results:** Of the 125 patients, 102 (81.6%) and 23 (18.4%) were assigned to the success and failure groups, respectively. According to the univariate analysis, the absence of anisometropia, a smaller preoperative near exodeviation, a better near stereopsis, a smaller magnitude of deviation on day 1 postoperatively, and response to patching were significantly associated with surgical success for IXT after 1 year. In the multivariate analysis, distant esotropic deviation on day 1 postoperatively and response to patching were the factors affecting successful surgical outcomes. **Conclusions:** Esotropic distant deviation on day 1 postoperatively is a prognostic factor for favorable surgical outcomes. Preoperative patching could be a factor influencing surgical success in children with IXT.

## 1. Introduction

Intermittent exotropia (IXT) is characterized by periods of alternating normal binocular alignment with sensory fusion and a manifest exotropia [1,2,3]. The pathophysiology of IXT has not been clearly defined. However, most current theories are based on the concept that the exodeviations are caused by a combination of mechanical and innervational factors. Innervational factors consist of variations in convergence innervation or a disturbed equilibrium between convergence and divergence [4]. The contributing factors to IXT include neuro-mechanical factors [5], insufficient fusion, a high accommodative convergence to accommodation ratio (AC/A) [6], refractive errors [7], and genetic factors [8], as cited in previous reports.

The optimal method for treating IXT has not yet been established, with both surgical and nonsurgical treatment options commonly prescribed [9,10]. The purpose of treatment is to improve the alignment and control of both eyes, thereby preserving binocular vision and stereopsis and preventing further loss. The non-surgical treatment of IXT includes patch therapy, prism therapy, orthoptic sessions, and overcorrecting minus lens therapy. The objective of these treatments is to reduce the symptoms and frequency of manifest deviation by decreasing the angle of deviation or enhancing the ability to control it [11]. Occlusion therapy has been employed as an anti-suppression therapy to stimulate motor fusion [12,13]. The potential benefits of occlusion include the preservation of binocularity and a reduction in the frequency or amount of exodeviation. Preoperative patching can improve binocularity and fusion ability, resulting in favorable surgical outcomes.

In addition to improving alignment and stereopsis, strabismus surgery can improve the psycho-social development of individuals, boosting their confidence and improving their quality of life [14]. The general criteria for surgical treatment include a reduction in or loss of stereoacuity (nearby or distant), deteriorating fusional control, a large angle of deviation, or a combination of these symptoms; however, the potential thresholds remain poorly defined [9]. Surgical treatment has the advantage of an immediate effect. However, it is limited in terms of cost and the risk of complications, including recurrence and overcorrection, which can also induce defects in fusion and stereopsis. Furthermore, while rare, vision-threatening complications such as retinal detachment, scleral perforation, and endophthalmitis can occur following surgery [5].

Many studies have been conducted on exotropia recurrence after surgery. As the success rate of surgery varies among studies (32.8–83.3%) [15,16,17], an exact prediction of surgical outcomes is difficult. This variation may be due to the differences in the definitions of surgical success, follow-up duration, patient populations, and surgical procedures. Numerous studies have suggested various factors that could be related to surgical success, including age at onset, timing of surgery [18,19,20], preoperative angle of deviation [16,20,21], visual acuity, type of surgery [21], type of exotropia [22], and stereopsis [23]. However, there is a paucity of information on the effectiveness of preoperative patching in maintaining successful outcomes after surgery for IXT [24,25].

Thus, the aim of this study was to determine the clinical features, including the response to preoperative patching, associated with the surgical outcomes in patients with IXT at 1 year postoperatively.

## 2. Materials and Methods

We retrospectively reviewed the medical records of 125 patients aged from 3 to 10 years old who underwent bilateral lateral rectus recession (BLR) surgery between January 2019 and February 2021, with >12 months of postoperative follow-up. Patients with constant exotropia, a history of ocular surgery, A-V pattern strabismus, oblique muscle dysfunction, vertical strabismus, paralytic strabismus, restrictive strabismus, unilateral amblyopia, nystagmus, or systemic diseases such as cerebral palsy were excluded from this study. The study protocol adhered to the Declaration of Helsinki tenets and was approved by the Institutional Review Board of Pusan National University Yangsan Hospital (IRB number: 05-2023-115). Since this was a retrospective study, the need for informed consent from the subjects was waived by the IRB of Pusan National University Yangsan Hospital.

### 2.1. Clinical Measurements

Patient records were reviewed to confirm the assessments of best corrected visual acuity (BCVA), stereoacuity, office-based control score, ocular alignment, suppression test, and cycloplegic refraction at the first visit. The charts of all patients with IXT who underwent surgical treatment during the time interval at one hospital were reviewed. Patients with missing information were excluded.

A visual acuity test was conducted for every patient with correction of refractive errors. The refractive errors were measured using cycloplegic refraction. The magnitude of exodeviation was measured using the alternate prism and cover test (APCT) at distance (6 m) and near (1/3 m) with accommodative target fixation.

Distant stereoacuity was assessed using the Distant Randot test at 3 m. Near stereoacuity was assessed using the Randot Preschool Stereo Test and the Titmus Stereo Fly Test at 40 cm (cm). Worth’s four-dot test was performed at 3 m and 33 cm to measure suppression or alternate fixation at distance and near. During the assessment of stereoacuity, control score, APCT, and suppression, the patients were corrected for BCVA.

Patients were prescribed patch treatment for 2 h/day(in addition to refractive correction, if needed). Patients who freely alternated between fixations were allowed to switch eyes on alternate days. Patching for 2 h per day on the dominant eye was prescribed if there was a strong fixation preference.

### 2.2. Strabismus Surgery

All patients in this study underwent bilateral lateral rectus recession (BLR) under general anesthesia, which was performed by one surgeon. Surgical dosages were applied using the modified Wright’s method, derived from the Parks surgical table based on the patient’s angle of deviation [26]. Patients with the convergence insufficiency type of exotropia underwent slanted BLR. The upper muscle fibers were recessed based on the distant angle of deviation, and the lower muscle fibers were recessed based on the near angle of deviation.

### 2.3. Outcome Measures

Surgical outcomes were grouped according to the angle of deviation at 1 year postoperatively as follows: success (esodeviation less than or equal to 5 prism diopters (PD) to exodeviation less than or equal to 10 PD) and failure (esodeviation more than 5 PD or exodeviation more than 10 PD) [27,28].

Patch responders were defined by one of the following criteria at a 3-month visit after the patching started: (1) decrease in the magnitude of exodeviation of 10Δ or more at distance and near compared to baseline, (2) improvement control score of ≥1 at distance or near, or (3) gain of stereoacuity of 2 octaves (0.6 log arcsec) or more at distance or near [29].

### 2.4. Factor Associated with Outcomes after Surgery

We investigated various clinical factors associated with surgical outcomes, including sex, visual acuity, refractive errors, presence of anisometropia, age at surgery, type of exotropia, stereopsis, duration from diagnosis to surgery, magnitude of exodeviation preoperatively, and magnitude of exodeviation on day 1 postoperatively.

Anisometropia was defined as the difference of hyperopia >+1.5 D, myopia >−1.5 D, and/or astigmatism >+1.5 D. Patients were classified as having basic IXT if the difference between the distance and near exodeviation was within 10Δ. The divergence excess (DE) type was defined when the distance deviation was 10Δ greater than the near deviation. The patient was classified as pseudo-DE type when they exhibited a greater exotropia at distance than near but the difference between the two decreased to less than 10Δ after 60 min of monocular occlusion. Convergence insufficiency (CI) was defined when the near deviation was 10Δ greater than the distance deviation.

### 2.5. Statistical Analysis

Pearson’s chi-square test was used to analyze categorical variables. An independent Student’s *t*-test was used to compare continuous variables. Before performing the *t*-tests, normality checks were performed. Outcome group (success vs. failure) comparisons were adjusted for the baseline level of the outcome using analysis of covariance. All *p*-values were obtained using two-tailed tests. SPSS (version 20.0; SPSS Inc., Chicago, IL, USA) was used for all analyses.

A logistic regression analysis was performed to identify factors associated with surgical outcomes. Each variable was analyzed using a univariate model. The significant variables were evaluated using multivariate models. Odds ratios (ORs) and confidence intervals (CIs) were reported. Statistical significance was set at *p* < 0.05.

### 2.6. Data Availability

The datasets generated during or analyzed during the current study are available from the corresponding author on request.

## 3. Results

The numbers of male and female patients were 55 (44.0%) and 70 (56.0%), respectively. The mean age was 5.7 ± 3.5 years at diagnosis and 7.5 ± 3.0 years at surgery. The exotropia type was basic in 106 (84.8%) patients, pseudo-DE type in 15 (12.0%), and convergence insufficiency type in 4 (3.2%). Sixty-six (66/125, 52.8%) patients were patch responders as defined in the Section 2 (Table 1).

Of the 125 patients, 102 and 23 belonged to the success and failure groups, respectively. The overall success rate was 81.6%. There were no differences in sex, age at diagnosis, age at surgery, time from diagnosis to surgery, BCVA, refractive errors, or type of exotropia in patients with surgical success versus failure. There were significantly more anisometropes (*p* = 0.04) and those with larger exodeviations (*p* = 0.04) in the failure group. The success group had significantly more patch responders (*p* = 0.04), those with good near stereopsis (*p* = 0.048) (Table 2), and those with distant and near esotropic deviation on day 1 postoperatively (*p* < 0.001) than the failure group (Table 3).

Absence of anisometropia (odds ratio [OR], 3.77; *p* = 0.038), smaller exodeviation at near (OR, 0.95; *p* = 0.036), good Titmus stereo test (OR, 0.47; *p* = 0.049), good response to patching (OR, 2.91; *p* = 0.037), and esotropic deviation at postoperative 1 day (OR, 0.778, *p* < 0.001 at distance; OR, 0.920, *p* < 0.001 at near) were significantly associated with successful outcomes at 1 year postoperatively according to the univariate analyses. The multivariate analysis revealed that patients with distant esotropic deviation on day 1 postoperatively (odds ratio [OR], 0.792; *p* = 0.004) and those who responded well to preoperative patching (OR, 4.356; *p* = 0.041) had successful surgical outcomes (Table 4).

## 4. Discussion

This study analyzed the surgical outcomes after BLR in 125 children diagnosed with IXT. Successful surgical outcomes were achieved in 81.6% of the children after one year of follow-up. Although direct comparisons are difficult due to differences in specific parameters and criteria used in each study, success rates have been reported to be approximately 32.8–83.3% in studies of similar standards [15,16,17,30,31]. Generally, studies with shorter follow-up periods reported higher rates of success than did those with longer follow-up periods.

To date, the factors that could possibly influence surgical outcomes have been well studied. In our study, it was found that the absence of anisometropia, smaller preoperative exodeviation at near, mild esotropic deviation on day 1 postoperatively, and good response to patching were associated with surgical success in patients with IXT.

The long-term results of the surgical treatment of IXT according to the type of surgery are controversial. A recent Pediatric Eye Disease Investigator Group (PEDIG) study reported no difference in the success rates between RR and BLR within the first 3 years after surgery [32]. Some reported that the success rate of the unilateral recession–resection (RR) group was significantly higher than that of the BLR group starting from the fourth post-operative year [27]. This study included only patients who had undergone BLR surgery for IXT to exclude the effect of the surgical method.

Scott et al. [33] reported that the status of refraction and degree of anisometropia were prognostic factors. In this study, there were significantly more anisometopes in the failure group. Since amblyopia can affect the recurrence of IXT [34], patients with amblyopia were excluded to prevent the effect of amblyopia on recurrence after surgery. Although bilateral visual acuity is similar, the quality of vision may differ from that of anisometropia. Thus, it may interfere with postoperative fusion and binocularity in patients with IXT, resulting in poor surgical outcomes.

Gezer et al. [35] reported that preoperative deviation was the most important factor in determining favorable outcomes in patients whose exotropia was treated with surgery and that patients with larger preoperative deviations had a poorer chance of successful outcomes. In the present study, the preoperative deviation at near was significantly smaller, and stereopsis based on the Titmus stereo test was better in the success group. Better fusional ability and stereopsis could be factors affecting maintenance after surgical IXT treatment.

Kim and Choi [28] conducted a study analyzing patients with IXT observed for more than 2 years after surgery and reported that the angle of deviation at distance and near on day 1 postoperatively was more esotropic in the success group than in the recurrence group; the mean deviation angle at postoperative day 1 in the success group was −5.34 (esodeviation) ± 5.92 PD at distance and −3.96 ± 5.87 PD at near, and in the recurrence group, it was −2.55 ± 4.81 PD at distance and −1.80 ± 5.87 PD at near (*p* = 0.000, *p* = 0.006). In the surgical management of exotropia, an initial overcorrection is widely agreed to be needed for satisfactory correction because of the tendency towards postoperative exotropic drift [28,36,37]. Raab [36] reported that an overcorrection of 10–20 PD provides the best outcome, although good outcomes were also obtained with overcorrections in the range of 0–10 PD. Lee and Lee [37] reported that a postoperative day-1 overcorrection of 11–20 PD following BLR surgery and 1–10 PD following R&R surgery can lead to good results. In the present study, the esotropic distant deviation on day 1 postoperatively was correlated with surgical success at 1 year postoperatively.

Our study showed that the surgical outcomes were favorable in patients who responded to preoperative part-time occlusion therapy, which is the most commonly used conservative treatment for IXT. Shin et al. [24] have suggested that preoperative part-time occlusion therapy is a possible factor in improving long-term surgical outcomes. Our previous study reported improvements in the angle of deviation, control scores, and stereoacuity at distance and near after part-time occlusion therapy [29]. Bang [38] reported a significantly higher surgical success rate in the improved control grade group. Suh [39] reported that after 3 months of occlusion therapy for 3 h each day, near-deviation measurements decreased significantly in both basic and convergence-insufficiency types of intermittent exotropia, indicating that fusional ability is increased with part-time occlusion therapy. Therefore, we hypothesized that patching can improve surgical results because it can improve fusional ability in intermittent exotropes.

This study had several limitations. First, as this was a retrospective study, the inclusion criteria might have been imprecise, and the follow-up period was uneven. Second, there may be potential selection and information biases. Patients with satisfactory results were less likely to return to the clinic. Conversely, those showing unfavorable results were more likely to have been followed up for longer periods. Third, when measuring the effect of patching, the parameters used in this study, such as the magnitude of exodeviation, improvement in control score, and stereoacuity, may vary depending on each child’s level of cooperation on the day of measurement, which could have led to bias. Fourth, there was no control group that did not receive occlusion treatment before surgery; thus, it was difficult to prove that preoperative patching was effective. Fifth, the study included a specific age group (3 to 10 years) of patients who underwent BLR surgery, which is appropriate for assessing pediatric strabismus. However, the extensive exclusion criteria may limit the generalizability of our findings to a wider pediatric population with varied strabismus characteristics. Finally, the definition of surgical success and failure based on postoperative deviation angles is clear, but may not fully capture the functional or quality-of-life outcomes that are important for pediatric strabismus management.

## 5. Conclusions

This study retrospectively explored the surgical outcomes of patients who received patching therapy before IXT surgery. Preoperative absence of anisometropia, good near stereoacuity, smaller exodeviation at near, and response to patching might be prognostic factors for favorable surgical results in patients with IXT. The multivariate analysis was performed to control for other confounding factors and revealed that preoperative patching improved surgical outcomes and that postoperative day 1 esotropic deviation and response to patching were associated with better surgical results.

## Figures and Tables

**Table 1:** Characteristics of patients with exotropia.

Variable	
Gender (M:F)	55:70
Age at diagnosis, mean ± SD (years)	5.7 ± 3.5
Age at operation, mean ± SD (years)	7.5 ± 3.0
Time from diagnosis to surgery, mean ± SD (years)	1.7 ± 2.0
Best corrected visual acuity (logMAR)	
OD, mean ± SD	0.03 ± 0.12
OS, mean ± SD	0.04 ± 0.12
Refractive errors in right eye, mean ± SD (diopters)	
Spherical	−0.19 ± 1.48
Cylinder	−0.75 ± 0.88
Spherical equivalent, SE	−0.56 ± 1.52
Refractive errors in left eye, mean ± SD (diopters)	
Spherical	−0.13 ± 1.40
Cylinder	−1.20 ± 4.49
Spherical equivalent, SE	−0.73 ± 2.77
Anisometropia, *n* (%)	12 (9.6)
Type of exotropia, *n* (%)	
Basic	106 (84.8)
Pseudodivergence excess	15 (11.2)
Convergence insufficiency	4 (3.2)
Stereopsis at near (logarcsec)	
Titmus Stereo Test	2.2 ± 0.6
Randot Stereo Test	1.9 ± 0.3
Stereopsis at distant	
Distance Randot Stereo Test	2.2 ± 0.3
Worth 4-dot test	
Suppression, *n* (%)	41 (37.27)
Alternate fixation, *n* (%)	14 (12.72)
Preoperative patching	
Good response, *n* (%)	66 (52.8)

**Table 2:** Preoperative and postoperative magnitude of deviation.

Magnitude of Deviation	
Preoperative deviation (PD)	
At distance, mean ± SD	29.7 ± 8.5
At near, mean ± SD	28.5 ± 10.5
Postoperative deviation on day 1 (PD)	
At distance, mean ± SD	−6.7 ± 6.4
At near, mean ± SD	−3.6 ± 6.0

Exotropia was noted as a positive value, and esotropia as a negative value.

**Table 3:** Association between demographics and surgical outcomes in patients with exotropia.

	Success Group (*n* = 102)	Failure Group (*n* = 23)	*p*-Value
Gender (M:F)	43:59	12:11	0.38 *
Age at diagnosis, m ± SD (years)	5.6 ± 3.2	6.2 ± 4.9	0.59 ^†^
Age at surgery, m ± SD (years)	7.3 ± 2.6	8.4 ± 4.3	0.21 ^†^
Time from diagnosis to surgery, m ± SD (years)	1.6 ± 2.0	2.2 ± 1.9	0.34 ^†^
BCVA (logMAR)			
OD, mean ± SD	0.03 ± 0.12	0.02 ± 0.11	0.535 ^†^
OS, mean ± SD	0.04 ± 0.11	0.04 ± 0.14	0.904 ^†^
Refractive errors in right eye, mean ± SD (diopters)			
Spherical	−0.18 ± 1.55	−0.22 ± 1.15	0.907
Cylinder	−0.67 ± 0.79	−1.11 ± 1.16	0.094
Spherical equivalent, SE	−0.52 ± 1.58	−0.78 ± 1.27	0.461
Refractive errors in left eye, mean ± SD (diopters)			
Spherical	−0.18 ± 1.47	0.10 ± 1.03	0.370
Cylinder	−1.25 ± 4.95	−0.99 ± 1.05	0.804
Spherical equivalent, SE	−0.81 ± 3.02	−0.39 ± 1.12	0.513
Anisometropia, *n* (%)	7 (6.8)	5 (21.7)	0.04 *
Type of exotropia			0.38 *
Basic	87	19	
Pseudodivergence excess	13	2	
Convergence insufficiency	2	2	
Stereopsis at near (logarcsec)			
Titmus stereo test	2.1 ± 0.5	2.4 ± 0.8	0.048 ^†^
Randot stereo test	1.9 ± 0.3	2.0 ± 0.3	0.586 ^†^
Stereopsis at distant			
Distance Randot stereo test	2.2 ± 0.3	2.3 ± 0.4	0.98
Worth 4-dot test			
Suppression, *n* (%)	31 (30.4)	9 (39.1)	0.62 *
Alternate fixation, *n* (%)	10 (9.8)	4 (17.4)	
Preoperative magnitude of deviation (PD)			
At distance, mean ± SD	29.2 ± 8.0	32.0 ± 10.3	0.15 ^†^
At near, mean ± SD	27.4 ± 9.8	33.1 ± 12.6	0.04 ^†^
Postoperative deviation on day 1 (PD)			
At distance, mean ± SD	−8.3 ± 5.0	0.3 ± 7.3	<0.001
At near, mean ± SD	−4.7 ± 5.4	1.6 ± 5.7	<0.001
Preoperative patching			0.04 *
Good response to patching, *n* (%)	53 (52.0)	13 (56.5)	

* Comparison between groups by Student *t*-test. ^†^ Comparison between groups by Pearson's chi-square test.

**Table 4:** Logistic regression analysis of the predictive factors associated with successful outcomes of bilateral lateral rectus surgery for intermittent exotropia.

Variable	Univariate Analysis			Multivariate Analysis		
	OR	95% CI	*p*-Value	OR	95% CI	*p*-Value
Gender (female)	0.668	0.270–1.656	0.384			
Age at diagnosis	0.956	0.847–1.079	0.466			
Age at operation	0.889	0.774–1.020	0.092			
Time from diagnosis to surgery	0.876	0.150–1.073	0.200			
BCVA (logMAR)						
OD	3.551	0.067–188.829	0.532			
OS	1.282	0.024–69.215	0.903			
Absence of anisometropia	3.770	1.076–13.202	0.038 *	0.417	0.059–2.954	0.381
Type of exotropia			0.498			
Basic						
Pseudodivergence excess	0.763	0.158–3.694	0.737			
Convergence insufficiency	0.167	0.014–1.963	0.154			
Stereopsis at near						
Titmus Stereo Test	0.471	0.223–0.996	0.049 *	1.207	0.360–4.052	0.761
Randot Stereo Test	0.981	0.149–6.453	0.984			
Stereopsis at distance						
Distance Randot Stereo Test	0.974	0.154–6.160	0.978			
Preoperative angle of deviation (PD)						
At distance, mean ± SD	0.964	0.915–1.015	0.159			
At near, mean ± SD	0.951	0.909–0.994	0.026 *	0.959	0.890–1.034	0.275
Angle of deviation on postoperative day 1 (PD)						
At distance, mean ± SD	0.778	0.699–0.867	<0.001	0.775	0.652–0.922	0.004 *
At near, mean ± SD	0.920	0.704–0.891	<0.001	0.886	0.812–1.198	0.986
Patch responders	2.908	1.013–8.348	0.047 *	4.356	1.064–17.836	0.041 *

* *p* < 0.05.

## Data Availability

The datasets generated or analyzed during the current study are available from the corresponding author upon reasonable request.
